# Anthelmintic Activity of Yeast Particle-Encapsulated Terpenes

**DOI:** 10.3390/molecules25132958

**Published:** 2020-06-27

**Authors:** Zeynep Mirza, Ernesto R. Soto, Yan Hu, Thanh-Thanh Nguyen, David Koch, Raffi V. Aroian, Gary R. Ostroff

**Affiliations:** 1 Program in Molecular Medicine, University of Massachusetts Medical School, Worcester, MA 01605, USA; zeynep.mirza@umassmed.edu (Z.M.); Ernesto.Soto-Villatoro@umassmed.edu (E.R.S.); yhu@worcester.edu (Y.H.); tthnguyen040@gmail.com (T.-T.N.); dkoch.79@gmail.com (D.K.); raffi.aorian@umassmed.edu (R.V.A.); 2 Department of Biology, Worcester State University, Worcester, MA 01602, USA

**Keywords:** terpenes, essential oils, yeast particles, anthelmintic, hookworm, whipworm

## Abstract

Soil-transmitted nematodes (STN) infect 1–2 billion of the poorest people worldwide. Only benzimidazoles are currently used in mass drug administration, with many instances of reduced activity. Terpenes are a class of compounds with anthelmintic activity. Thymol, a natural monoterpene phenol, was used to help eradicate hookworms in the U.S. South circa 1910. However, the use of terpenes as anthelmintics was discontinued because of adverse side effects associated with high doses and premature stomach absorption. Furthermore, the dose–response activity of specific terpenes against STNs has been understudied. Here we used hollow, porous yeast particles (YPs) to efficiently encapsulate (>95%) high levels of terpenes (52% *w/w*) and evaluated their anthelmintic activity on hookworms (*Ancylostoma ceylanicum)*, a rodent parasite (*Nippostrongylus brasiliensis*), and whipworm (*Trichuris muris)*. We identified YP–terpenes that were effective against all three parasites. Further, YP–terpenes overcame albendazole-resistant *Caenorhabditis elegans*. These results demonstrate that terpenes are broad-acting anthelmintics. Terpenes are predicted to be extremely difficult for parasites to resist, and YP encapsulation provides water-suspendable terpene materials without surfactants and sustained terpene release that could lead to the development of formulations for oral delivery that overcome fast absorption in the stomach, thus reducing dosage and toxic side effects.

## 1. Introduction

Soil-transmitted helminths or nematodes (STHs or STNs) are nematode parasites of the small intestine (hookworm and ascarids) and large intestine (whipworm) that pose enormous health problems for humans. Approximately 1–2 billion people are infected [1]. The effects of STN infections are most pronounced on children, as the infections can result in physical growth stunting, delayed intellectual development, cognitive impairment, anemia, malnutrition/lower nutritional status, school absenteeism, decreased future earnings, and immune defects that can lead to increased susceptibility to/virulence of malaria, tuberculosis, HIV/AIDS, and to vaccine failure [2,3,4,5]. Infections are also associated with an increased risk of maternal death, infant death, and low birth-weight babies in pregnant women [6] and with decreased adult worker health and productivity [7]. Thus, STN infections currently contribute significantly to trapping large populations in poverty worldwide.

Because of the scale of the problem, the treatment of STN infections in humans depends upon mass drug administration (MDA). Only four drugs, belonging to the benzimidazole class of compounds, are approved by the World Health Organization (WHO) for human STN therapy, and of these, only one drug, albendazole, has adequate efficacy for MDA [8,9]. Albendazole has good efficacy against *Ascaris*, moderate and variable efficacy against hookworms [10], and poor efficacy against whipworms. Recent data from MDAs have revealed instances of the unusually low efficacy or reduced efficacy of albendazole over time against hookworms, *Ascaris*, and whipworms [10,11,12,13,14,15], raising the specter of parasite resistance (already rampant in veterinary medicine [16]). Furthermore, after repeated annual rounds of MDA, the transmission indices can reach plateau levels, indicating that the complete elimination of STN parasites from the community cannot be achieved with the limited anthelmintics in hand [14,17,18]. New classes of anthelmintics with broad specificity, and that are more difficult to resist than current therapies, are urgently needed. 

Terpenes and terpenoids are naturally occurring compounds that constitute the primary component of essential oils obtained from plants, and have the potential to be used as anthelmintics. Terpenes (e.g., cymene, limonene, pinene) are hydrocarbons consisting of isoprene repeating units. Terpenoids (e.g., terpineol, carvacrol, thymol) are modified terpenes containing additional functional groups (usually oxygen-containing groups). In this paper, the term terpene is used to include both terpenes and terpenoids. These compounds have been long recognized for a wide range of functional properties, such as their insecticidal, antifungal, and antibacterial activity [19,20], and many terpenes are safe and approved for applications in food, cosmetics, and in the pharmaceutical industry. The use of terpenes has played an important role in traditional medicine worldwide, for example, medicinal plants such as Asian wormwood and American wormseed contain terpenes that are thought to confer their reported anthelmintic properties [21,22,23]. Thymol was successfully used to help eradicate hookworm infections in the United States in the early 1900s [19,24]. Although effective, the use of thymol as an anthelmintic has long been discontinued. Large doses are required, as >90% of orally administered thymol is rapidly absorbed in the stomach and proximal intestine, resulting in less than 10% reaching the target sites where STNs reside. These large doses are also associated with toxic side effects, including mucosal irritation and nausea [24,25]. This limitation of thymol and other terpene-based therapies might be overcome by using encapsulation techniques and enteric coated formulations to target effective terpene doses directly at the site of STN infections, thus minimizing adverse side effects. Additionally, despite the success of thymol as a cure for hookworms, little systematic work has been done to study the efficacy of specific terpenes against STNs.

A potential benefit of terpenes as an anthelmintic is that, given their generalized and multiple mechanisms of action evolved over the millennia as plant defense molecules, terpenes are extremely difficult to be resisted by target pathogens [26]. Screens for terpene-resistant variants have yet to uncover any significant resistance. For instance, in a forward genetic transposon screen for *E. coli* resistant to thymol, the best mutant barely increased the *E. coli* minimum inhibitory concentration (MIC) of thymol from 0.41 mM (wild type) to 0.53 mM (mutant) [27]. Another significant advantage of terpenes is their putative modes of action, worked out in their use as fungicides and bactericides. Terpenes have general membrane-disrupting capabilities, resulting in chemo-osmotic stress and the generalized death of their targets [28,29]. 

We have developed methods using yeast particles (YPs) to efficiently encapsulate high levels of terpenes. YPs are 3–5 μm hollow and porous microspheres, a byproduct of the food grade yeast (*Saccharomyces cerevisae*) extract manufacturing process. We have used yeast particles for the encapsulation of a broad range of molecules for drug delivery and agricultural applications [30,31,32,33,34,35,36]. 

In this article, we systematically studied the dose–response impact of terpenes on STN parasites. We screened 17 YP-encapsulated terpenes and three YP-encapsulated essential oil samples for in vitro activity against three parasitic worm species: the human hookworm parasite *Ancylostoma ceylanicum* (a major human health problem in Southeast Asia [10,37]), the murine whipworm *Trichuris muris* (a good model for human whipworm, *Trichuris trichuria* [38]), and the rat parasite *Nippostrongylus brasiliensis*, which is closely related to human hookworms. The results showed that terpenes vary in efficacy and can be categorized into five classes: (1) fast-acting and potent at moderate–high doses, (2) fast-acting at high doses, (3) slow-acting and potent at low doses, (4) slow-acting at moderate–high doses, and (5) non-effective at the doses tested. Importantly, active terpenes were equally effective against wild type and an albendazole-resistant *Caenorhabditis elegans* strain, suggesting that terpenes could improve treatment against drug-resistant nematodes.

## 2. Results 

### 2.1. Yeast Particle Encapsulation of Terpenes

**Figure 1:**
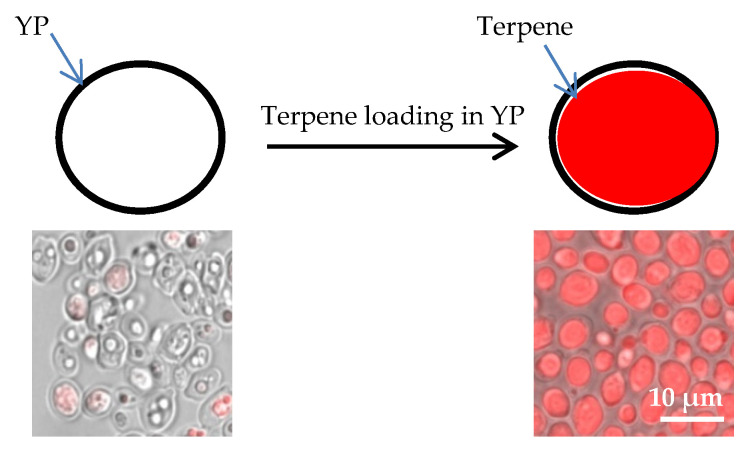
Schematic of diffusion-controlled terpene loading in yeast particles (YPs) and micrographs of samples stained with Nile red, showing empty YPs and YPs loaded with terpene (citral).

Yeast particles (YPs) are hollow, porous microparticles (3–5 μm) derived from baker’s yeast. The porous cell wall structure makes these particles excellent absorbent materials. Terpene encapsulation in YPs is based on the loading of terpenes inside the hydrophobic YP cavity by the passive diffusion of terpene through the porous cell walls in an aqueous suspension of YPs (Figure 1). We selected 17 low-cost, commercially available terpenes (chemical structures depicted in Figure S1 and key properties for YP loading and characterization are listed in Table S1) and three essential oils (lavender, tea tree, and peppermint oil) that contain several terpenes for encapsulation. A high loading capacity (1.1:1 *w/w* payload:YP) was achieved with all twenty target materials. Nile red dye was used to stain terpenes to qualitatively assess loading. The pictures in Figure 1 provide visual evidence of empty YPs and YPs fully loaded with a terpene (citral). Selected YP–terpene materials were extracted in 90% methanol–10% water to quantify terpene loading by HPLC. The HPLC results showed that terpenes were encapsulated with >98% efficiency (Table S2).

### 2.2. Anthelmintic Activity of YP–Terpenes In Vitro

The anthelmintic activity of YP–terpenes was evaluated in vitro against four species of worms in the adult stage: the human hookworm parasite *A. ceylanicum,* the murine whipworm *T. muris,* the rat parasite *N. brasiliensis,* and albendazole-resistant and wild-type *C. elegans*. All studies were carried out at terpene concentrations from 0 to 333 µg/mL. Free terpenes and empty YPs were also evaluated as controls. Empty YPs do not show toxicity on worms and active free terpenes require high concentrations (1 mg/mL) for effective toxicity on worms. All in vitro results below used YP–terpenes.

**Figure 2:**
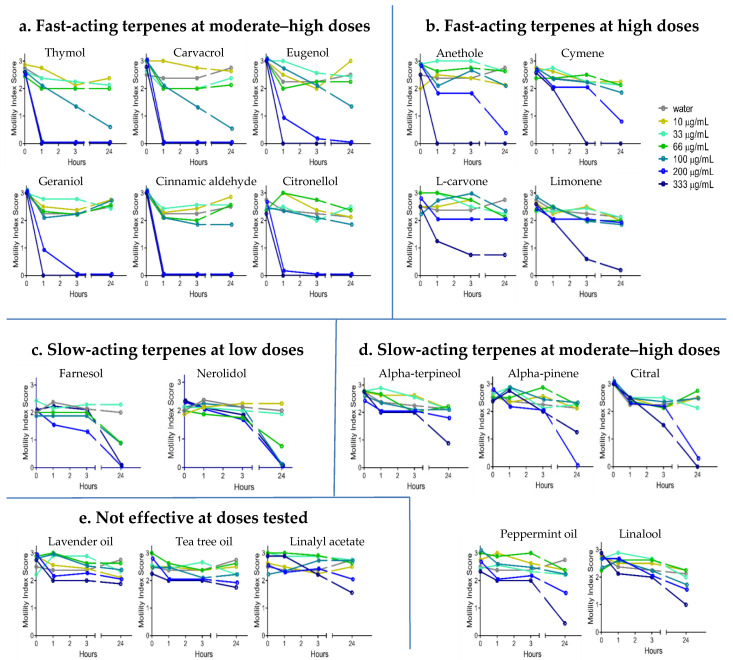
Dose–response of YP–terpenes on adult *Ancylostoma ceylanicum* in vitro, as shown for (**a**) fast-acting terpenes at moderate–high doses, (**b**) fast-acting terpenes at high doses, (**c**) slow-acting terpenes at lower doses, (**d**) slow-acting terpenes at moderate–high doses, and (**e**) terpenes not effective at the doses tested. Terpenes were evaluated at concentrations from 0 to 333 µg/mL. Parasitic worms were scored at the times indicated for motility, where 3 is actively moving, 2 is slow moving, 1 is not moving unless touched, and 0 is not moving even when touched (likely dead). N = eight adult worms/condition.

YP–terpene activity against *A. ceylanicum:* The hookworm screen results showed that the efficacy of YP–terpenes varies, and terpenes can be classified into five groups based on their dose–response curves (Figure 2). The first class of terpenes, which consists of thymol, carvacrol, eugenol, geraniol, cinnamic aldehyde, and citronellol, was very effective at inactivating hookworms at moderate–high (200–333 μg/mL) doses in one to three hours. Yet, 100 μg/mL doses of these terpenes did not show activity in the same timeframe of 1 to 3 h. A second group of terpenes, consisting of anethole, cymene, limonene, and L-carvone, were effective only at high doses (333 μg/mL) at the three-hour time point.

The results for the other groups are as follows: The third group of terpenes, which consists of farnesol and nerolidol, were not effective in one to three hours; however, these terpenes killed the worms at lower doses (66 μg/mL) in 24 h. The fourth group of terpenes, which consists of alpha pinene, citral, alpha terpineol, linalool, and peppermint oil, showed activity only at the 24-h time point at moderate–high doses. Finally, linalyl acetate, tea tree oil, and lavender oil did not show any activity against hookworms at the doses tested. 

**Figure 3:**
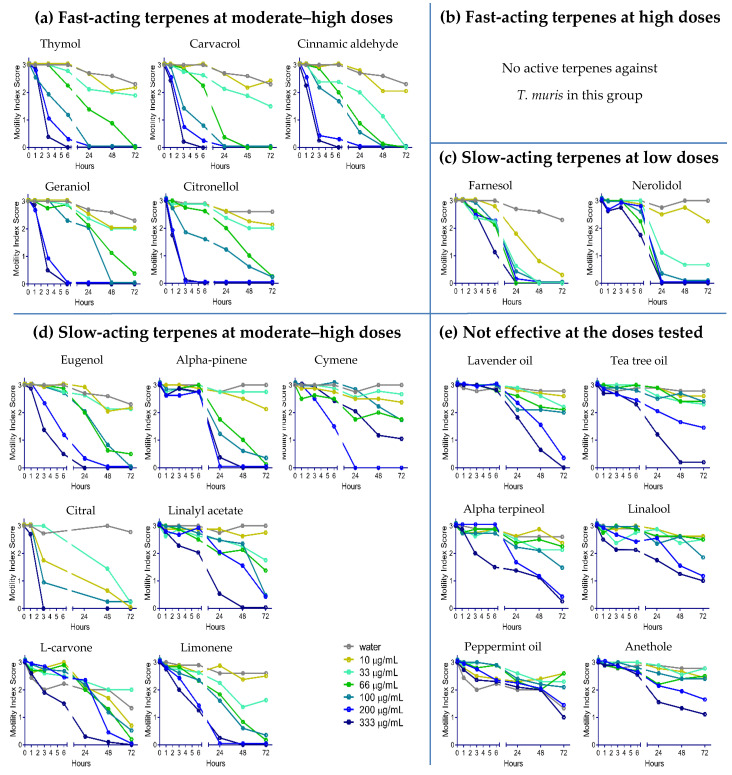
Dose–response of YP–terpenes on adult *Trichuris muris* in vitro, as shown for (**a**) fast-acting terpenes at moderate–high doses, (**b**) fast-acting terpenes at high doses, (**c**) slow-acting terpenes at lower doses, (**d**) slow-acting terpenes at moderate–high doses, and (**e)** terpenes not effective at the doses tested. Terpenes were evaluated at concentrations from 0 to 333 µg/mL. Parasitic worms were scored at the times indicated for motility, where 3 is actively moving, 2 is slow moving, 1 is not moving unless touched, and 0 is not moving even when touched (likely dead). N = eight adult worms/condition.

YP–terpene activity against *T. muris:* The same YP–terpene samples were screened against *T. muris* (Figure 3). Overall, *T. muris* was less susceptible to terpenes than *A. ceylanicum* (minimum effective terpene doses against each worm are summarized in Table 1). The fast-acting terpenes at moderate–high doses against *A. ceylanicum*, with the exception of eugenol, also immobilized *T. muris* within between one and three hours. Eugenol, cymene, limonene, L-carvone, alpha pinene, citral, and linalyl acetate were effective at moderate–high doses at the 24-h time point. As seen with *A. ceylanicum*, farnesol and nerolidol acted slowly against *T*. *muris* but required low doses. Lastly, we did not observe any anthelmintic activity with alpha-terpineol, linalool, anethole, peppermint oil, lavender oil, or tea tree oil against *T. muris.*

**Figure 4:**
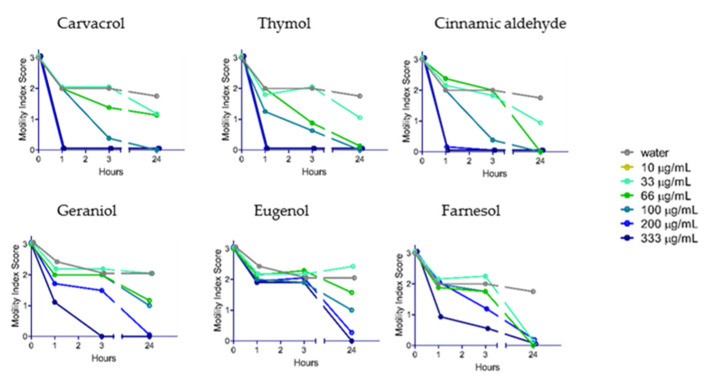
Effect of select YP-terpenes on *N. brasiliensis.* N = eight adult worms/condition.

YP–terpene activity against *N. brasiliensis*: After testing the terpenes on *A. ceylanicum* and *T. muris*, we screened selected YP–terpenes on *N. brasiliensis* (Figure 4). The dose–response curves were similar to those obtained with *A. ceylanicum* and *T. muris*. While carvacrol, thymol, and cinnamic aldehyde immobilized *N. brasiliensis* worms within one hour at 333 and 200 μg/mL doses, geraniol and eugenol were less effective. Farnesol was still effective at low doses at the 24-h time point.

**Figure 5:**
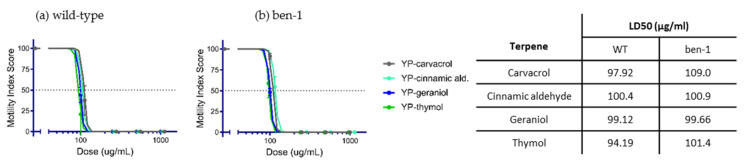
Dose–response curves and LD50 values of fast-acting YP-terpenes on (**a**) wild-type (WT) and (**b**) albendazole *ben-1(e1880*)-resistant *C. elegans*. N = 20 L4–adult worms per condition scored at 6 days.

YP-terpene activity against *C. elegans:* Terpenes classified as fast-acting at moderate–high doses in the hookworm and whipworm screening were evaluated on wild-type and albendazole-resistant *C. elegans* to assess whether these terpenes can overcome albendazole *ben-1(e1880*) resistance. The results showed that LD50 values were very similar for each terpene on wild-type and *ben-1(e1880*) worms (Figure 5), indicating that the mechanism of action of these terpenes is different than albendazole. 

### 2.3. Kinetics of Terpene Release from YPs

**Figure 6:**
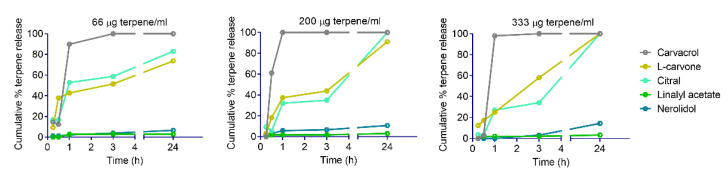
Cumulative terpene release from YP-terpene samples diluted in water at terpene concentrations of 66, 200, and 333 μg/mL.

Terpene release from YPs is based on passive diffusion out of the particles and is a function of terpene water solubility. YP–terpene samples were diluted at different terpene concentrations in water, and the terpene release from particles into the supernatant was quantified at different timepoints by HPLC. The graphs of cumulative terpene release, as a function of time at three different dilutions in water (66, 200, and 333 μg/mL), are shown in Figure 6 for terpenes representative of each group classified based on anthelmintic activity. Carvacrol, a fast-acting terpene with a maximum solubility in water of 1.3 mg/mL, shows a fast release (>90% at 1 h) from YPs at all concentrations. L-carvone has a similar solubility in water as carvacrol, but it shows a slower rate of release from YPs (40–60% at 3 h) and almost complete terpene release at 24 h. The lipid content in YPs is likely to impact the release of terpenes upon dilution in water, as shown in the different release patterns of two terpenes (carvacrol and l-carvone) with similar solubility in water. This reduction in terpene release correlates with the experimental observation that L-carvone is fast-acting on nematodes only at high concentrations. Terpenes that are slow-acting at high concentrations (e.g., citral) show a slow release profile from YPs (30–40% at t = 3h at 200 and 333 μg/mL). Terpenes that do not show anthelmintic activity also do not show significant terpene release from YPs (e.g., linalyl acetate). The terpenes in this group have very low solubility in water (<10 μg/mL) and remain trapped in the hydrophobic YPs. Finally, two highly water insoluble terpenes (farnesol and nerolidol) were classified as slow-acting at low doses. The analysis of nerolidol release from YPs shows a small increase in nerolidol eluting from YPs over time, with up to 10–15% released at 24 h. The amount of nerolidol released from YPs is 20–30 times higher than its maximum solubility in water, suggesting that this terpene is eluting from the particles as an oil-in-water emulsion. 

### 2.4. Difference in Uptake of YP–Terpenes by Hookworms and Whipworms.

**Figure 7:**
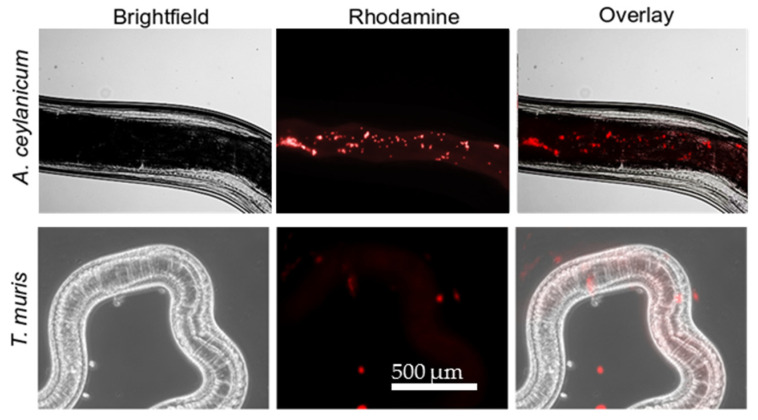
Microscopy pictures showing the ingestion of YPs by hookworms, but not whipworms, after a 24 h incubation with rhodamine-labeled YPs.

Hookworms and whipworms showed differential susceptibility to YP–terpenes. We hypothesized that the difference in terpene activity between these two parasite species might be explained by YP–terpenes acting with two different mechanisms: (1) terpene release from YP–terpenes to the medium, followed by terpene absorption by the worms and (2) YP–terpene ingestion by worms and subsequent terpene release inside the worms. Rhodamine-labeled YPs (rYPs) were used to evaluate YP uptake by worms and the results after a 24 h incubation show that *A. ceylanicum* avidly eats rYPs (Figure 7). These results support that the YP–terpene effect on *A. ceylanicum* proceeds by two mechanisms, but YP–terpene activity on *T*. *muris* is limited to only terpene release from YPs to the medium. 

## 3. Discussion 

Although terpenes have been used in humans as anthelmintics for more than 100 years, there is a surprising dearth of careful research in this area. Here, we systematically look at the dose–response efficacy of a wide range of terpenes against a phylogenetically diverse range of STNs. The potential major advantages of terpenes are: (1) they are broad acting, (2) they are predicted to be extremely difficult for parasites to resist, and (3) their pharmacodynamics may be vastly improved by modern formulation strategies (e.g., encapsulation and enteric coated capsules to protect premature release and absorption in the stomach) that are predicted to overcome their traditional limitations with regards to unpleasant taste and toxicity. 

The entrapment of hydrophobic terpenes in YPs occurs by passive diffusion through the YP shell into its hydrophobic interior, forming a terpene oil droplet filling the hollow cavity inside the YP (Figure 1). Terpene encapsulation in YPs offers several major advantages for in vitro testing: (1) high terpene load levels (50% terpene *w/w*), (2) water-suspendable terpene formulations without surfactants, (3) high terpene bioavailability, and (4) sustained terpene release. 

The dose–response screening of YP–terpenes in vitro against the hookworm *A. ceylanicum* (Figure 2) and the whipworm *T. muris* (Figure 3) show that the terpenes may be grouped into five pharmacodynamic classes: (1) fast-acting terpenes at moderate–high doses (100–333 μg/mL), (2) fast-acting at high doses (333 μg/mL), (3) slow-acting at lower doses (33–66 μg/mL), (4) slow-acting at moderate–high doses (100–333 μg/mL), and (5) not effective at the doses tested.

Most fast-acting terpenes have a solubility in water higher than the dose tested, allowing for rapid terpene release from YPs in 1–3 h (e.g., carvacrol, Figure 6). Terpenes like L-carvone also have a high solubility but the kinetics of release are slow, likely due to terpene–lipid interaction in YPs, and as result of the slower rate of terpene release, L-carvone is fast-acting only at high doses. Terpenes in the slow-acting at a moderate–high dose group have a low water solubility, requiring a longer incubation time (≥ 24 h) to achieve an effective terpene dose to release from YPs. Interestingly, terpenes in the slow-acting at a low dose group are extremely water insoluble, but we found out that terpenes in this group (farnesol, nerolidol) self-emulsify in YP–terpene aqueous suspensions, releasing the terpene at rates 20–30 times higher than its maximum solubility in water. 

Overall, *T. muris* was less susceptible to terpenes than *A. ceylanicum*. For example, the fast-acting terpenes at moderate–high doses showed an effect on *A. ceylanicum* in 1 h at 200 μg/mL, but it took a slightly longer time (3 h) for these terpenes to be active on *T. muris* at the same concentration. Additionally, we did not identify any terpenes in the second group (fast-acting at a high dose) against *T. muris*. Relative to *A. ceylanicum*, more terpenes were slow-acting at moderate–high doses against whipworm, or not even effective at all. 

The screening results of selected YP–terpenes on *N. brasiliensis* (Figure 4) show that this hookworm species is susceptible to all the selected terpenes with carvacrol, thymol, and cinnamic aldehyde, showing complete worm incapacitation in 1 h at 200 µg/mL. This rat parasite is closely related to human hookworms, making it a convenient intestinal nematode/rodent laboratory model that we will use in future studies to test the efficacy of orally administered YP–terpenes. 

To determine if terpenes are able to overcome anthelmintic resistance, we treated the free-living laboratory nematode *Caenorhabditis elegans*, both wild-type and albendazole-resistant genotypes, with YP–terpenes (Figure 5). Both strains are fully susceptible to all terpenes tested.

Our results demonstrate the possibility of using YP–terpenes as a high efficacy pan-anthelmintic therapeutic with the potential to overcome resistance. Future work depends upon marrying traditional plant-based medicines that were effective, but have been long out of favor, with modern formulation and therapeutic technologies that have the potential to overcome the drawbacks of these “old-school” therapies (e.g., bad taste, premature absorption in the stomach, and mucosal irritancy) that historically limited their use. Surprisingly, purified terpenes, as a broad class, have not been probed in great breadth or depth against STNs. To our knowledge, there has also been no systematic attempt at using the very best and modern formulation technologies to allow terpenes to reach their full, and likely vast, therapeutic potential against STNs. 

## 4. Materials and Methods 

YPs were purchased from Biorigin (Louisville, KY, USA). Terpenes and HPLC solvents were obtained from Thermo Fisher Scientific (Waltham, MA, USA) or Sigma-Aldrich (St. Louis, MO, USA). Essential oils were obtained from the following suppliers: tea tree oil (*Melaleuca alternifolia*) from Vitacost (Lexington, NC, USA), peppermint oil (*Mentha piperita L.)* from Laboratoire I.R.I.S. (Eygluy-Escoulin, France), and lavender oil (*Lavandula angustifolia)* from Custom Essence (Somerset, NJ, USA). Reagents for worm culture medium were purchased from Gibco (Gaithersburg, MD, USA). 

### 4.1. YP Loading of Terpenes

Dry YPs were mixed with water (180 g YP/L) and the slurry was passed through an Emulsiflex^®^-C3 high-pressure homogenizer (Avestin, Ottawa, ON, USA) to obtain a uniform single YP suspension. Samples of homogenized YPs (0.9 g dry YPs) were mixed with terpene (990 mg) and incubated at room temperature with the exception of YP–limonene (40 °C) and YP–thymol (60 °C). Samples were incubated for a minimum of 24 h to allow for complete terpene loading. Samples were stained with Nile red to assess loading by the fluorescence microscopy of the encapsulated terpene–Nile red complex. YP–terpene samples (100 mg) were centrifuged to collect excess liquid (free terpene) and the pellet fraction was resuspended in 10 mL of 90% methanol–10% water to extract the encapsulated terpene. Both free and encapsulated terpene fractions were quantified by HPLC operated with 32 Karat^TM^ software version 7.0 (Beckman Coulter, Inc, Brea, CA, USA), using a Waters Symmetry^®^ C18 column (3.5 μm, 4.6 × 150 mm) with acetonitrile:water 70:30 as a mobile phase, flow rate at 1 mL/min, injection volume of 10 μL, and terpene detection at 254 nm. This isocratic HPLC method allows the detection of single terpene samples with terpene retention times varying from 2.7 to 18.5 min. The quantification of terpenes was done by measuring the peak area and interpolating the concentration using a calibration curve obtained with terpene standards. For the analysis of the three essential oils, the peak of the main terpene in the oil was used for quantification. All samples were run in triplicate. 

### 4.2. Terpene Release from YP–Terpenes

YP–terpene samples were resuspended in water and incubated at room temperature. Aliquots were collected at predetermined times, centrifuged, and the supernatant collected to measure the terpene released from the particles by HPLC. 

### 4.3. Maintenance of Parasites and C. elegans

*A. ceylanicum* worms were maintained in golden Syrian hamsters, as previously described [39]. *T. muris* parasites were maintained in STAT6-/-mice [40]. *N. brasiliensis* were maintained in Wistar rats [41]. *C. elegans* strains were cultured as per standard protocols [40]. Ethical approval: All animal experiments were carried out under protocols approved by the Institutional Animal Care and Use Committee (IACUC) of the University of Massachusetts Medical School (UMMS protocol number A-2483, A-2484, A-2518). Hamsters, mice, and rats used in this study were housed, handled, fed, and experimentally used following the National Institute of Health (NIH) Guide for the Care and Use of Laboratory Animals in Research. Euthanasia was performed by CO_2_ asphyxiation, followed by bilateral pneumothorax.

### 4.4. Adult Worm In Vitro Screening

Adult worms were harvested from infected rodents and washed using prewarmed medium (RPMI 1640 with 25 mM HEPES (pH 7.2)) and antimicrobials (100 U/mL penicillin, 100 µg/mL streptomycin, 1 µg/mL fungizone). Eight worms were manually picked into each well of the 24-well screening plate containing 500 μL RPMI per well. As serum is incompatible with the assays with YP–terpenes, we left it out. Hence, this limited the duration of the in vitro motility assays with hookworms. YP–terpene samples were evaluated at concentrations of 0 to 333 μg terpene/mL. Assay plates were incubated at 37 °C and 5% CO_2_. Terpene activity was determined at different timepoints using the standard motility index [42]. A motility index of 3 was given to active worms, 2 for slow-moving worms, 1 for motile after stimulation by touching, and 0 for not moving even when touched (likely dead). 

## 5. Conclusions

Yeast particles were used to efficiently encapsulate twenty different terpenes and essential oils, and the dose–response of YP–terpenes was evaluated against the human-infecting zoonotic hookworm, *A. ceylanicum*, and the murine whipworm *T. muris* (a good model for human whipworm). This is the most systematic examination of a wide range of terpenes against STNs. The anthelmintic activity of YP–terpenes was evaluated based on the average motility index after 1–3 h and 24 h (hookworm) or 24–72 h (whipworm). Five main groups of terpenes can be observed based on anthelmintic activity, fast-acting terpenes at moderate–high doses, fast-acting at high doses, fast-acting at low doses, slow-acting terpenes at moderate–high doses, slow-acting terpenes at low doses, and finally the terpenes that were not effective at the doses tested. Carvacrol, thymol, and cinnamic aldehyde were consistently fast-acting at moderate–high doses on *A. ceylanicum* and *T. muris*. Eugenol was fast-acting against *A. ceylanicum* but slow-acting against *T. muris*. Anethole, cymene, limonene, l-carvone, alpha-terpineol, linalool, peppermint oil, and linalyl acetate fell into different categories for *A*. *ceylanicum* and *T. muris* and they were less effective for the latter parasite. Alpha-pinene and citral were consistently slow-acting at moderate–high doses against both parasites. Similarly, farnesol and nerodiol exhibited similar killing kinetics—slow but effective at low doses—on *A. ceylanicum* and *T. muris*. The difference in the activity of terpenes on the two worm species can be explained at least partly by YP–terpenes acting on *A. ceylanicum* hookworms by two mechanisms: terpene release from YPs into the medium followed by absorption by worms and a second mechanism of direct YP–terpene ingestion by hookworms. In contrast, *T. muris* whipworms do not ingest YPs and the terpene mode of action on this whipworm depends only on terpene release from YPs into the medium. 

We extended our adult worm in vitro screen with lead candidates to the rat hookworm parasite *N. brasiliensis,* and albendazole-resistant and wild-type *C. elegans.* The results show that the selected terpenes (carvacrol, thymol, cinnamic aldehyde, farnesol) have broad anthelmintic activity and have the potential to be developed into anthelmintic drugs. Our next step will be to evaluate this in vivo using the *N. brasiliensis*/rat model to target lethal terpene doses in the intestine using enteric coated YP–terpenes or using YP-encapsulated acid-resistant biodegradable pro-terpene drugs [35] to avoid the premature release of terpene from YPs.

## Figures and Tables

**Table 1:** Minimum effective doses and classification of YP–terpenes against *A. ceylanicum* and *T. muris*.

	*A. ceylanicum*			*T. muris*		
**Terpene**	**1 h**	**3h**	**24 h**	**1 h**	**3 h**	**24 h**
Carvacrol	200	200	200	333	200	66
Cinnamic aldehyde	200	200	200	333	200	66
Thymol	200	200	100	>333	200	100
Geraniol	200	200	200	>333	200	100
Citronellol	200	200	200	>333	200	200
Eugenol	200	200	200	>333	>333	200
Anethole	333	333	200	>333	>333	>333
Cymene	>333	333	200	>333	>333	333
Limonene	>333	333	333	>333	>333	200
L-carvone	>333	333	333	>333	>333	333
Farnesol	>333	>333	66	>333	>333	33
Nerolidol	>333	>333	66	>333	>333	66
Alpha pinene	>333	>333	200	>333	>333	200
Citral	>333	>333	333	>333	-	200
Alpha terpineol	>333	>333	333	>333	>333	>333
Linalool	>333	>333	333	>333	>333	>333
Peppermint oil	>333	>333	333	>333	>333	>333
Linalyl acetate	>333	>333	>333	>333	>333	333
Lavender oil	>333	>333	>333	>333	>333	>333
Tea tree oil	>333	>333	>333	>333	>333	>333

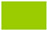
 Fast acting at moderate-high doses 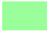 Fast acting at high doses 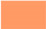 Slow acting at low doses 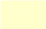 Slow acting at moderate-high doses 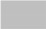 Not effective at doses tested.

## Supplementary Materials

The following are available online, Figure S1: Chemical structure of terpenes, Table S1: Octanol/water partition coefficient (log P), solubility in water, and HPLC retention time with isocratic method (acetonitrile:water 70:30) of terpenes evaluated for encapsulation in YPs, Table S2: Terpene loading of selected YP–terpene samples.
