# Supplementary material for: Anthelmintic Activity of Yeast Particle-Encapsulated Terpenes
Source: Molecules. 2020 Jun 27;25(13):2958. doi: 10.3390/molecules25132958 (PMC7411854; doi:10.3390/molecules25132958)
Supplement: Supplementary file 1 [file molecules-25-02958-s001.pdf]

Supplementary material

Anthelmintic Activity of Yeast Particle-Encapsulated Terpenes

Zeynep Mirza, Ernesto R. Soto, Yan Hu, Thanh-Thanh Nguyen, David Koch,
Raffi V. Aroian and Gary R. Ostroff,*

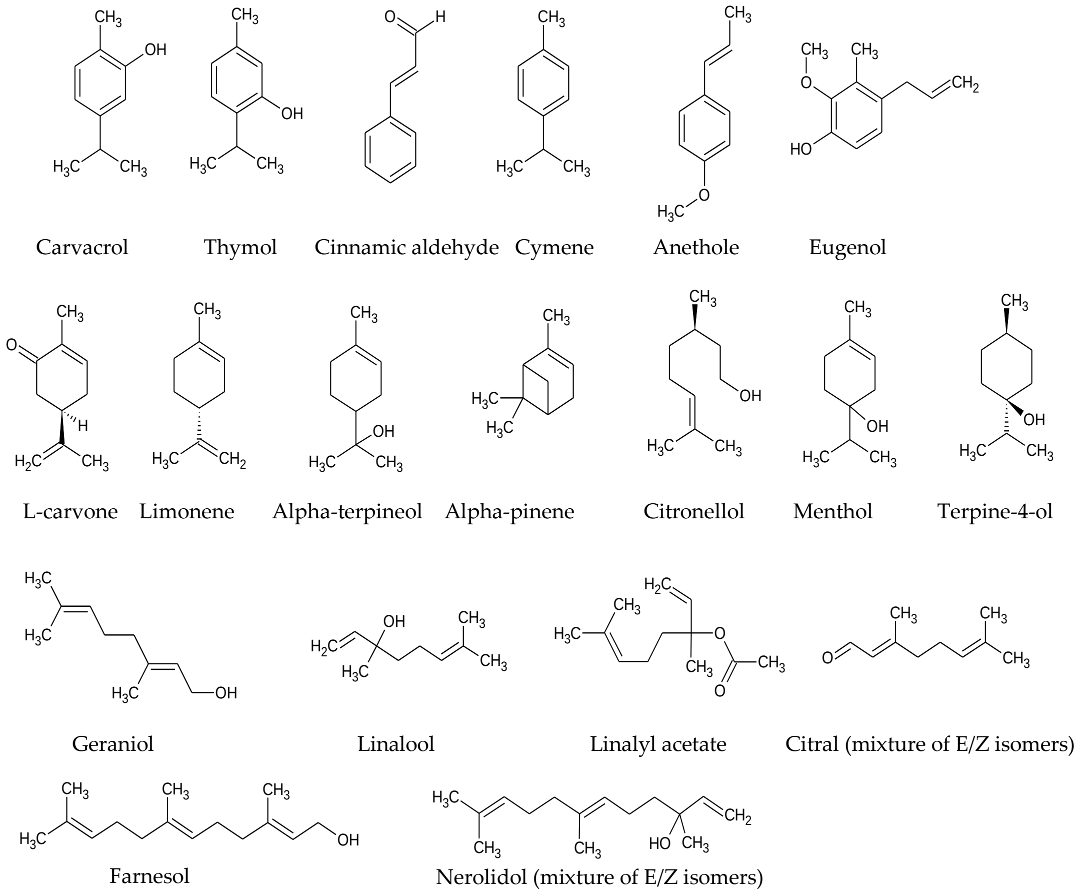

Figure S1. Chemical structure of terpenes.

Table S1. Octanol/water partition coefficient (log P), solubility in water, and HPLC retention time with isocratic method (acetonitrile:water 70:30) of terpenes evaluated for encapsulation in YPs.

Terpene		Log P	Solubility in Water	HPLC Retention Time (Min)
Cinnamic aldehyde		1.9	1.42 mg/mL	2.63
Eugenol		2.27	1.44 mg/mL	2.73
L-carvone		2.71	1.31 mg/mL	3.35
Citral		2.76	0.59 mg/mL	3.87
Linalool		2.97	1.6 mg/mL	3.48
Alpha terpineol		2.98	7.1 mg/mL	3.38
Thymol		3.3	0.9 mg/mL	3.42
Carvacrol		3.43	1.25 mg/mL	3.35
Geraniol		3.56	0.686 mg/mL	3.35
Citronellol		3.91	0.307 mg/mL	3.71, 4.32
Anethole		n/a	0.111 mg/mL	4.90
Linalyl acetate		3.93	8.2 μg/mL	6.61
Cymene		4.1	23.4 μg/mL	8.31
Limonene		4.57	7.57 μg/mL	8.1
Nerolidol		4.6	1.532 μg/mL	8.28
Alpha-pinene		4.83	2.49 μg/mL	18.47
Farnesol		5.7	1.7 μg/mL	10.6
Peppermint oil (menthol)*		3.4	0.42 mg/mL	8.40, 8.87
Tea tree oil (terpine-4-ol)*		2,81	2.5 mg/mL	9.9–10.4
Lavender oil*	Linalool	2.97	1.6 mg/mL	3.48
	Linalyl acetate	3.93	8.2 μg/mL	6.61

*These oils are complex mixtures, for example, lavender oil contains over 100 terpenes. The properties of the primary terpenes found in these oils are shown.

Table S2. Terpene loading of selected YP–terpene samples.

Terpene	HPLC Retention Time (Min)	HPLC Limit of Detection (μg/mL)	% YP-Encapsulated Terpene
Carvacrol	3.35	8	98.5 ± 2.5
L-carvone	3.35	8	99.7 ± 0.1
Citral	3.87	4	99.8 ± 0.1
Cymene	8.31	8	99.8 ± 0.2
Linalyl acetate	6.61	8	100.0 ± 0.1
Nerolidol	8.28	8	100.0 ± 0.5
